# Michigan’s Peril

**Published:** 1900-02

**Authors:** 


### MICHIGAN'S PERIL.

On another page in this issue will be found a defence of the State veterinary law of Michigan and an answer to an editorial comment appearing in the Journal.

What those who are most familiar with legislative action in behalf of the profession have learned to fear has more than come to pass in Michigan.

The law not only encourages the maintenance of two-year colleges, but makes the way easy for future enterprises in this direction. We say enterprises, because we know that many of our private veterinary institutions are simply one-man schools, and are used to enhance the personal interests of a single individual. These schools cannot advance save by and through the consent of their owner or promoter. They must be made self-supporting, as private enterprises are not endowed, as a rule, and are not in a position to command national, State, or municipal aid or support. This practical question of sustenance must be met, and there are many times when it is done at great peril to the integrity of the college.

Such laws solve but few of the ends desired, and if, as in Michigan, they become fire-brands in the profession, setting up a prairie-fire-like conflagration, destroying the unity, harmony, and worth of the profession, better would it have been had such a law never been enacted. Its experience in angry litigation, its threats and demands of the profession are already so discordant as to never again harmonize, and the wounds and sores left after but a few months' existence are not likely to heal while the present generation lives. The broken ranks, the bitter invectives used, and the personal animosities engendered will live for many years, and no act that will add one iota of strength to the profession is yet visible as the result of Michigan's legislative effort. Such are the painful lessons learned, and, while it is only history repeating itself, it seems strange that what has been learned in other States from like bitter experiences was not a warning to others. We sincerely trust that the fire now burning so furiously in Michigan may burnish her escutcheon so bright and free it from the stains and tarnishes that she will prove the safety gate of experience to her sister States. The American Veterinary Medical Association can do much in aiding our colleagues in their dilemma, and every effort should be made to accomplish this much-to-be-desired end.

